# Self-Reported Visual Difficulty and Mortality Risk Among Older Adults: The Mediating Role of Recurrent Falls

**DOI:** 10.1093/geroni/igaf016

**Published:** 2025-02-17

**Authors:** Shu Xu, Jeffrey A Burr, Qian Song, Joshua R Ehrlich

**Affiliations:** Department of Gerontology, University of Massachusetts Boston, Boston, Massachusetts, USA; Institute for Social Research, University of Michigan, Ann Arbor, Michigan, USA; Department of Gerontology, University of Massachusetts Boston, Boston, Massachusetts, USA; Department of Gerontology, University of Massachusetts Boston, Boston, Massachusetts, USA; Institute for Social Research, University of Michigan, Ann Arbor, Michigan, USA; Department of Ophthalmology and Visual Sciences, University of Michigan, Ann Arbor, Michigan, USA

**Keywords:** Death, Discrete-time survival models, Vision loss

## Abstract

**Background and Objectives:**

Visual difficulty is associated with an increased risk of mortality, however the mechanisms accounting for the link between visual difficulty and mortality have not been described. This study examined the association between visual difficulty, recurrent falls, and mortality risk among Americans aged 65 and older.

**Research Design and Methods:**

This longitudinal study employed data from the *2015–2020 waves of the National Health and Aging Trends Study* (*N* = 7,039). Visual difficulty was assessed by questions regarding self-reported near and distance vision and the use of visual aids. Recurrent falls were defined as experiencing a fall more than one time within the last year. Discrete-time survival models with a structural equation modeling-based approach were estimated for the associations between visual difficulty, recurrent falls, and all-cause mortality.

**Results:**

Among the respondents, 8.1% reported visual difficulty at baseline. Compared to those without visual difficulty, older adults with visual difficulty were more likely to experience recurrent falls. Having visual difficulty at baseline was associated with experiencing recurrent falls in the following wave (β (log-hazard odds) = 0.12, *p* < .001) and with mortality in subsequent waves (β = 0.39, *p* < .001). The association of visual difficulty with mortality was mediated by recurrent falls (β = 0.05, *p* < .001).

**Discussion and Implications:**

Compared to those with normal vision, older adults with visual difficulty are more likely to experience recurrent falls, which may in turn increase the risk of mortality. Future research should investigate other potential pathways to gain a more complete understanding of the relationship between visual difficulty and mortality risk.

**Translational Significance:** Visual difficulty is associated with an increased risk of mortality; however, the mechanisms accounting for the link between visual difficulty and mortality remain unclear. Our study highlights the longitudinal association between visual difficulty and mortality among older adults, as well as the mediating role of recurrent falls in this association. Older adults with visual difficulty and anyone involved in their care should be aware of the risks of recurrent falls. A thorough assessment of their living environment and personal needs is crucial to identify gaps in support and explore ways to improve safety in this population.

Vision loss is a common condition among older adults, and vision loss has been associated with an increased risk of mortality (Ehrlich et al., 2021; Zhang et al., 2016). Although research has begun to assess the effect of vision loss on mortality through multiple health pathways (Christ et al., 2008; Zheng et al., 2012), the likely mechanisms accounting for the link between vision loss and mortality risk are still unclear, including the role that falls may play in this association. Compared to those with normal vision, individuals with vision loss are more likely to fall, suffer recurrent falls, and experience a higher risk of fall-related fractures (Freeman et al., 2007; Legood et al., 2002).

Falls are also a leading cause of injury and mortality among older adults, with evidence demonstrating that older adults who experience falls have an increased risk of mortality (Jónsdóttir & Ruthig, 2021; Nurmi et al., 2004; Padrón-Monedero et al., 2020; Sylliaas et al., 2009). Vision loss among older adults can increase the risk of falls, which in turn may result in serious injuries that can increase the risk of mortality. Thus, the purpose of this study is to investigate the relationship between self-reported visual difficulty (termed “visual difficulty” hereafter) and all-cause mortality, including examining falls as one pathway linking visual difficulty and mortality.

## Vision Loss and Mortality Risk

Adults with disabilities have a higher risk of mortality than adults without disabilities, and this association is significant for most types of disabilities and most causes of death (Forman-Hoffman et al., 2015). Studies have explored the relationships between different types of disability, functional status, mental illness, and mortality (Forman-Hoffman et al., 2015). These studies report that mobility limitations, substance use, and sensory disability had the strongest association with mortality (Forman-Hoffman et al., 2015). As people age, they may experience sensory loss, which can impose unique challenges, among them an increased risk of mortality (Schubert et al., 2017). The causes of age-related sensory dysfunctions are varied and complex. Some dysfunctions may be due to systemic factors that affect both sensory systems and mortality risk (Schubert et al., 2017). The most common causes of death in the United States among people 65 years and older are heart disease, cancer, chronic lower respiratory disease, stroke, Alzheimer’s disease, and diabetes (Heron, 2017). Many of these chronic health conditions have multiple risk factors, including subclinical atherosclerosis and inflammation that are also risk factors for vision loss (Klein et al., 2014).

As one of the most prevalent physical impairments among older adults, vision loss is increasingly found to be related to mortality risk (Ehrlich et al., 2021; Zhang et al., 2016). In general, most studies find that vision loss is significantly associated with an increased risk of all-cause mortality. A meta-analysis of 29 prospective studies confirmed this relationship, indicating that adults with vision loss had a significantly higher risk of mortality compared to adults without vision loss (Zhang et al., 2016). In a recent systematic review and meta-analysis conducted by Ehrlich and colleagues (2021), the authors found a dose–response relationship, wherein greater degrees of vision loss were associated with a higher hazard of all-cause mortality. Emerging research assesses the effect of vision loss on mortality with potential moderators and mediators, including gender, physical activity, functional status, allostatic load, mental well-being, and preventive care practices (Christ et al., 2008; Zheng et al., 2012). However, studies that examine the potential pathways in the vision loss-mortality relationship are limited.

## The Mediating Role of Falls

Existing evidence supports the relationship between vision loss and falls (Dhital et al., 2010), as well as falls and mortality risk (Padrón-Monedero et al., 2020). However, few studies focus on the mediating role of falls in the association between vision loss and mortality risk. Vision problems have been identified as an intrinsic risk factor for falls (Berg & Cassells, 1992).

Reduced visual functions including impaired visual acuity, contrast sensitivity, glare sensitivity, stereoacuity, and visual field size, were significantly associated with increased risk of falls (Mehta et al., 2022; Saftari & Kwon, 2018). Decreased vision may affect balance control, distract attention from surroundings, reduce one’s ability to detect hazards in the environment, and therefore place an individual at greater risk of falls (Buckley et al., 2010). People with vision loss may lack stability when stepping up and down stairs or when adopting different gaits when trying to avoid obstacles, both of which can increase the risk of tripping and falling (Buckley et al., 2010). As a large proportion of falls occur due to slipping, tripping (Lord & Dayhew, 2001), and interacting with environmental hazards (Rubenstein, 2006), vision loss has emerged as an essential factor when considering fall risk.

Older adults with vision loss are at high risk of falling. Various visual functions such as visual acuity, contrast sensitivity, glare sensitivity, and visual field size typically decline with age (Saftari & Kwon, 2018). Anatomical changes in the eyeball and increased aberrations due to aging contribute to the deterioration of spatial vision, adversely affecting the quality of visual inputs to the central nervous system and overall visual performance (Saftari & Kwon, 2018). Moreover, age-related eye diseases like cataracts, age-related macular degeneration (AMD), and glaucoma are significantly linked to an increased risk of falls (Saftari & Kwon, 2018). Vision loss may impair balance control and increase cognitive load, reducing the ability to multitask (Buckley et al., 2010; Sloan et al., 2005). Compared to the general older population, preventing falls and promoting safe locomotion are especially challenging for older adults with vision loss (Brundle et al., 2015; Ramulu et al., 2019). Many older adults with vision loss need to allocate more attention to maintaining their balance during everyday activities, thus experiencing a higher risk of falling (Buckley et al., 2010). When dealing with substantial challenges in navigating their surroundings, especially those with bilateral vision loss, older adults may develop a fear of falling and experience activity limitations. These limitations can lead to musculoskeletal issues, deteriorations in gait and balance, and ultimately, an increased risk of falling (Gupta et al., 2023). The impact of vision loss on getting around the home and on negotiating the external environment is identified as one of the five risk factors that have the strongest association with falling among older adults (Brundle et al., 2015). Nearly half of older adults with severe visual difficulty or blindness fell in the prior year, whereas over a quarter of those without severe visual difficulty fell during this same time period (Crews et al., 2016).

Falls are one of the most common causes of injury among older adults and can result in significant mortality risk; falls are one of the leading causes of death among older adults (James et al., 2020). Research shows that two-thirds of unintentional injuries, which can lead to death, are caused by falls (Rubenstein, 2006). Mortality related to falls has been on the rise among older adults, with an increasing age-adjusted trend observed between 2007 and 2016 (Burns & Kakara, 2018; Centers for Disease Control and Prevention, 2024). Given the rapid aging of the population, this trend is expected to continue. Empirical studies also confirm that older adults who experience falls have an increased risk of mortality compared to those who do not experience falls (Jónsdóttir & Ruthig, 2021; Padrón-Monedero et al., 2020; Sylliaas et al., 2009). Moreover, social determinants of health play a critical role in the risk of falls and subsequent mortality, especially among individuals with vision loss. Factors such as limited access to eye care, lower income, and reduced educational attainment are associated with vision loss and exacerbate the challenges faced by older adults, further increasing their risk of falls and mortality (Hudak et al., 2023).

In sum, the current scientific literature emphasizes the importance of falls for negative health outcomes, including mortality. Among older adults who survive the acute impacts of a fall, falls in later life continue to predict subsequent survival over the following years (Nurmi et al., 2004). To add further insight into the overall burden of falls, longitudinal research on mortality using panel data is needed, particularly among populations with greater risk of falls, such as older adults with vision loss. This study contributes to the scientific literature by examining the mediating role of falls in the visual difficulty-mortality association.

## The Stress Process Model

The stress process model developed by Pearlin and colleagues focuses on three conceptual domains, including the source of stressors, factors that mediate or moderate the effects of stress, and health outcomes (Pearlin et al., 1981, 2005). The stress process model has served as an influential framework for understanding mechanisms by which stressors lead to health outcomes (Aneshensel & Mitchell, 2014). According to the stress process model, major life events, chronic strains, daily hassles, and traumas challenge individuals’ coping skills. In turn, these experiences create new stresses that threaten to overwhelm people both physically and mentally, increasing the risk of illness (Pearlin & Bierman, 2013). The effects of stressors on health outcomes also depend on the extent to which stressors proliferate.

The stress process model has been empirically supported in a large number of studies and has been applied across a wide range of populations (Folkman, 2012). However, to the best of our knowledge, this model has never been applied to understanding the process through which people respond to visual difficulty in later life outcomes. The stress process model has the advantage of being directly relevant to older adults with visual difficulty and offering an explanation of how visual difficulty, directly and indirectly, is associated with mortality. Visual difficulty can be conceptualized as a source of stress, which may negatively affect health outcomes, such as mortality risk. Visual difficulty may create new strains and increase the likelihood of falling. Falls can also be conceptualized as a source of stress that leads to worse health outcomes, such as a high mortality risk. According to the related idea of “stress proliferation” (Pearlin et al., 2005), falls, as the stressor that results from visual difficulty, may expand primary stressors (e.g., visual difficulty) and worsen outcomes (e.g., mortality risk). Thus, the objective of this study is to examine the association between visual difficulty and mortality risk among U.S. older adults, along with the mediating role of falls in this relationship over a 6-year observation period. Four hypotheses are proposed:

H1: Older adults with visual difficulty have a higher mortality risk compared to those with normal vision.
H2: Older adults with visual difficulty have a higher risk of recurrent falls compared to those with normal vision.
H3: Older adults who experience recurrent falls have a higher mortality risk compared to those who do not experience falls.
H4: The association between visual difficulty and mortality risk is mediated by recurrent falls.

## Method

### Data Source and Study Sample

**Figure 1.**
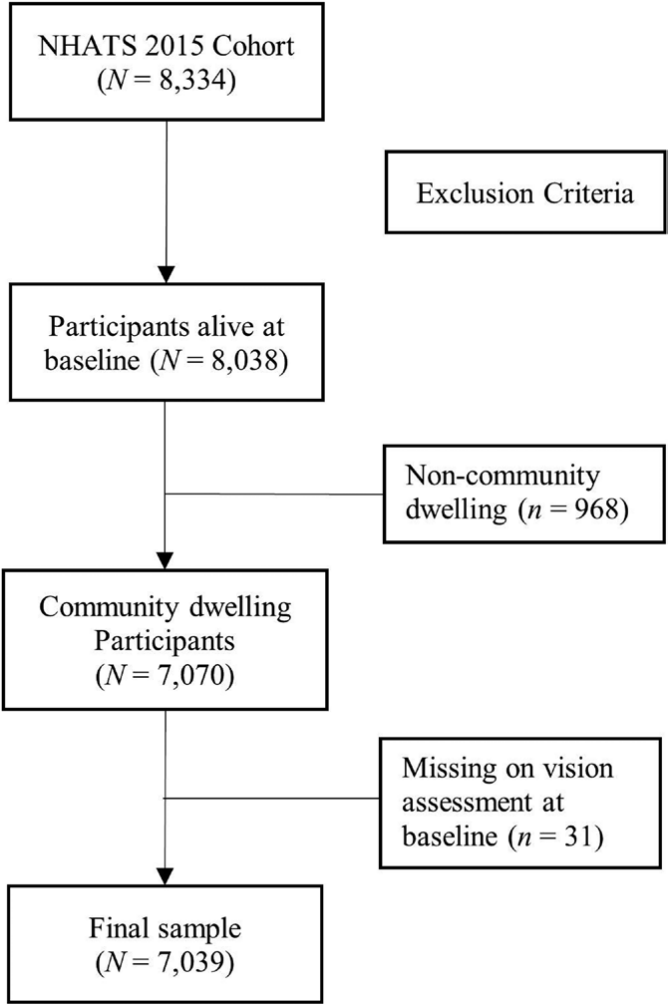
Sample size flow chart. NHATS = National Health and Aging Trends Study.

This study used data from the *National Health and Aging Trends Study* (NHATS), an ongoing nationally representative study of U.S. Medicare beneficiaries ages 65 and older with annual follow-up. The sample is drawn from the Medicare enrollment files using a stratified three-stage sample design. The initial sample was interviewed in 2011, with a sample size of 8,500 participants, and replenishment of the sample was undertaken in 2015 and 2021. The current longitudinal study included participants from the 2015 NHATS cohort. The baseline sample of this cohort included 8,334 participants. Among these, 4,064 participants survived until 2020, 2,038 participants died, and 2,232 participants were lost to follow-up for other reasons over the 6-year interval. In this study, participants who were not community-dwelling or lacked information on visual difficulty at baseline were excluded, leaving a study sample of 7,039 participants (Figure 1).

### Measures

#### Dependent variable

Participants’ death was reported to NHATS personnel by informants during attempts to contact the participant for their annual follow-up interview. In situations where the participant did not respond to requests for the annual interviews, proxy informants were contacted for information on the status of the participant. These individuals confirmed whether the participant died. If a proxy was unavailable or did not provide mortality information, the participant was treated as missing and not included in the analysis. Information on the specific cause of death is not available in NHATS. In this study, we used year of death to assess all-cause mortality risk. Mortality status was measured as 0 = alive, 1 = deceased.

#### Independent variable

In NHATS, self-reported visual difficulty was assessed by asking questions regarding near and distance vision, along with the use of visual aids. Participants are considered to have visual difficulty if they reported blindness or were not able to see well enough to recognize someone across the street and/or were unable to read newspaper print when using glasses or contact lenses, as in prior studies using NHATS data (Patel et al., 2020). Visual difficulty was measured at baseline as 0 = no visual difficulty, 1 = visual difficulty.

#### Mediator

In the NHATS, falls are defined as “any fall, slip, or trip in which you lose your balance and land on the floor or ground or at a lower level” (Guralnik et al., 1995). Fall-related variables included falls within the last month and falls within the last year, along with the number of falls in the last year. Participants were first asked if they had fallen in the last month, if yes, they were asked if they had fallen *more than one time* within the last year; if not, they were asked if they had fallen within the last year, with a follow-up question about whether they experienced multiple falls within the last year. Compared to single-fall incidents, recurrent falls generally result in more serious consequences and are more concerning from an overall health perspective (Vaishya & Vaish, 2020). The American and British Geriatrics Societies recommend using a threshold of more than one fall in the past year to screen for fall risk (Panel on Prevention of Falls in Older Persons, American Geriatrics Society and British Geriatrics Society, 2011). Thus, for this study, falls were measured as 1 = recurrent falls in the last year and 0 = one or no falls in the past year.

#### Covariates

This study included a number of relevant demographic and health covariates, including age, gender, racial/ethnic status (non-Hispanic White, Hispanic, non-Hispanic Black, non-Hispanic other), education (<high school, high school degree/some college/undergraduate and graduate degree), marital status (married/living with partner, divorced/separated/widowed/never married), and a series of dichotomous indicators for self-reports of heart disease, hypertension, diabetes, lung disease, stroke, cancer, and a broken or fractured hip. The dementia status of participants was evaluated using (1) self-reported physician diagnosis of dementia, (2) the Alzheimer’s Disease-8 Screening interview, and (3) a cognitive test battery that evaluated participants’ memory, orientation, and executive function. The NHATS dementia classification algorithm was created by combining these three domains and categorizing participants into three groups: (1) probable dementia; (2) possible dementia; and (3) no dementia (Kasper et al., 2013). For this study, dementia status was measured as a dichotomous variable based on the NHATS classification: 0 = no dementia; 1 = possible or probable dementia. Body mass index (BMI) was calculated with participants’ height and weight and categorized as underweight, (<23 kg/m^2^), healthy weight (23–33 kg/m^2^), or overweight (>33 kg/m^2^) using standards (Winter et al., 2014) for older adults. Smoking status was coded as 1 = current smoker and 0 = never smoked or former smoker.

### Analytic Strategy

Descriptive characteristics of the study sample at baseline were presented. Bivariate analyses (*t* tests or chi-square tests) were used to compare participants who had visual difficulty and those without visual difficulty across the study variables at baseline. Discrete-time survival models (DTSM) with a structural equation modeling-based approach were used to test the hypotheses. The DTSM is useful for longitudinal studies when the data are collected at discrete-time periods and avoids the methodological issue of ties in the timing of the occurrence of events—death (Singer & Willett, 2003). In this study, participants were included until the proxy-reported death occurred or they were right-censored at the end of the study (did not experience death), whichever occurred first. The DTSM was estimated using a full-information maximum likelihood approach (Muthén & Masyn, 2005; Singer & Willett, 2003), which allowed for missing data in the form of noninformative right censoring, incorporating the partial information available about the event times for right-censored individuals into the likelihood function for the sample.

The DTSM also allowed for the examination of direct and indirect effects of predictors on the timing of death. The direct effect in the DTSM model captured the influence of a predictor on the hazard probability of event occurrence controlling for the mediator, and the indirect effect captured the influence of the predictor on the hazard probability of event occurrence through the mediator (McDaniel, 2018). In this study, the predictor (visual difficulty) was assessed at baseline (wave 1), the mediator (falls) was assessed at the next wave (wave 2), and the onset-to-event outcome (mortality) was assessed in subsequent waves (wave 3 to wave 6). Falls were regressed on visual difficulty to produce the path *a* estimate. A variable representing the latent propensity for the onset of the event (mortality) was created. The mortality risk latent variable was regressed on the mediator, falls, to yield the path *b* estimate. Finally, the mortality risk latent variable was regressed on visual difficulty to yield the direct effect—*c’* parameter estimate. The *a* and *b* parameter estimates were multiplied to determine the total indirect effect of *ab* (Fairchild et al., 2015). The DTSM model including a mediator with a time-variant effect was estimated using the logit link function for the time-to-event outcome (mortality). The model was estimated using MPlus Version 8, with a maximum likelihood estimator with robust standard errors (MLR).

**Figure 2.**
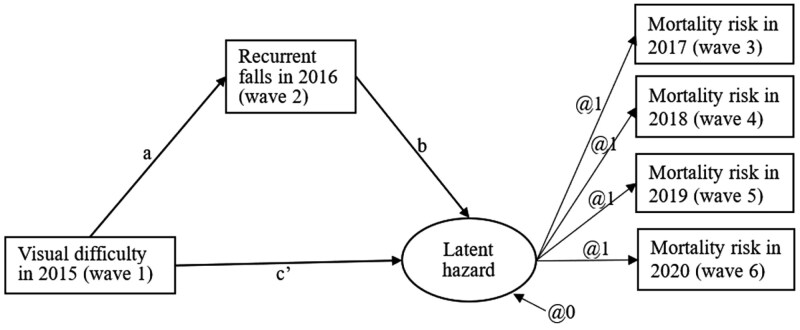
Discrete-time survival model results within the structural equation model framework for visual difficulty and mortality risk via recurrent falls.

The path diagram for the DTSM for visual difficulty, falls, and mortality risk is shown in Figure 2 using standard conventions of path models where observed variables are represented by rectangles, latent variables by circles, direct effects by straight arrows that point from cause to effect, and residuals by straight arrows pointing at the dependent variable. To specify a proportional odds model for mortality, a latent construct representing mortality risk, with mean and variance constrained to zero, was estimated with mortality information from wave 3 to wave 6, loading equally. Therefore, the raw coefficients are interpreted as capturing risk of mortality, measured in standard deviations, given a unit change in the explanatory variables. Since the discrete-time survival part of the model was interpreted in terms of a linear regression using the latent response formulation (Pratschke et al., 2016), the direct effects in the statistical model are equivalent to “natural direct effects,” while the total effect was given by the sum of the direct and indirect effects (Pearl, 2014). The size of the mediation effects is reported in Table 3, both in absolute terms and as a mediation proportion, with standard errors and confidence intervals (Ditlevsen et al., 2005).

**Table 3.** Effects of Visual Difficulty on All-Cause Mortality Risk Mediated Through Recurrent Falls

Variable	Log-hazard odds	*SE*	Est/*SE*	*p* Value
Direct effect				
Visual difficulty	0.392	0.100	3.935	.000
Indirect effects				
Visual difficulty—recurrent falls—mortality risk	0.047	0.012	3.764	.000
Total effect				
Visual difficulty—mortality risk	0.439	0.100	4.379	.000
Mediation proportion	0.106	0.035	3.009	.003

*Notes*: *N* = 7,039. EST = estimate; *SE* = standard error.

## Results

Descriptive characteristics of the weighted study sample at baseline are presented in Table 1. Among the participants, 8.1% had visual difficulty, 58.0% were aged between 65 and 74, 54.1% were female, 58.6% were married, 79.9% were non-Hispanic White, 33.0% had no degree or high school degree, 65.3% had healthy weight, and 9.3% were smokers. Moreover, 13.6% of the participants had hip fractures experienced recurrent falls in the previous year.

**Table 1.** Descriptive Characteristics for Study Sample and Bivariate Analyses by Visual Difficulty, NHATS 2015

Characteristics	Overall	Without visual difficulty	With visual difficulty	χ^2^
Visual difficulty, %	8.05			
Age groups, %				102.90^***^
65–69	30.44	30.98	24.25	
70–74	27.54	28.12	20.82	
75–79	19.06	19.05	19.20	
80–84	12.13	12.01	13.44	
85–89	7.33	6.82	13.13	
90+	3.51	3.02	9.15	
Female, %	54.10	53.45	61.83	15.96^**^
Married, %	58.59	57.50	45.36	69.17^***^
Race, %				
Non-Hispanic White	79.92	81.23	65.03	105.58^***^
Non-Hispanic Black	8.51	8.16	12.5	
Hispanic	7.57	6.72	17.33	
Non-Hispanic other	3.99	3.89	5.13	
Education, %				188.58^***^
No degree	17.08	15.41	36.21	
High school	25.90	25.62	29.11	
Some college	23.11	23.62	17.38	
College or above	33.90	35.36	17.30	
Comorbidities, %				
Heart disease	17.26	16.61	24.75	24.02^***^
Hypertension	65.56	64.58	76.72	33.94^***^
Stroke	5.78	5.35	10.70	27.25^**^
Dementia	14.69	12.81	36.06	294.67^***^
Lung disease	17.01	16.56	22.12	11.42^**^
Cancer	18.51	18.68	16.59	1.51
Hip fracture	2.37	2.12	5.20	18.43^***^
Body mass index, %				16.16^**^
Underweight	16.14	15.83	19.87	
Healthy weight	65.29	65.96	57.37	
Overweight	18.57	18.21	22.76	
Medicare dual eligibility, %	11.58	10.30	26.50	124.51^***^
Current smoking, %	9.29	9.37	8.37	0.46
Recurrent falls during the previous year (wave 2)	13.56	12.47	26.95	74.83^***^

*Notes*: *N* = 7,039. Statistics based on weighted data. NHATS =National Health and Aging Trends Study.

^*^ *p < *.05.

^**^ *p < *.01.

^***^ *p < *.001.

The bivariate results show that respondents with and without visual difficulty differed in terms of falls, sociodemographic characteristics, and specific chronic diseases. Older adults with visual difficulty were older, were more likely to be female and non-Hispanic White, had lower levels of education, and were less likely to be married. They were less likely to have a healthy weight, more likely to have hip fractures, and twice as likely to experience recurrent falls in the previous year. In addition, Supplementary Figure S1 presents Kaplan–Meier survival curves illustrating 5-year survival probabilities for groups with and without visual difficulties, further stratified by the presence of recurrent falls. Older adults with visual difficulty who experienced recurrent falls had the lowest survival probabilities, whereas those without visual difficulty and who did not experience recurrent falls had the highest survival rates.

Results from the DTSM are presented in Table 2 (direct effect results) and Table 3 (indirect effect results). All four of the study hypotheses received support. As shown in Table 2, regressing falls onto visual difficulty at baseline yielded an *a* path of 0.115 (.019), *p* < .001. These log-hazard odds indicated that there was a significant effect of visual difficulty on falls, such that having visual difficulty at baseline was associated with a 0.103 unit increase in recurrent falls in the following year. Regressing mortality risk onto visual difficulty and falls yielded parameters estimates of *c*’ = 0.422 (0.100), *p* < .001, and *b* = 0.392 (0.083), *p* < .001, respectively. The *c*’ path revealed that there was a significant effect of visual difficulty on mortality risk, controlling for falls; the *b* parameter revealed that falls significantly affect mortality risk in the subsequent waves, controlling for visual difficulty. For people with visual difficulty at baseline (wave 1), there was a greater likelihood of experiencing recurrent falls in the next wave (wave 2) and the likelihood of mortality increased in the subsequent waves (wave 3 to wave 6). For people who experienced recurrent falls (wave 2), the likelihood of mortality increased in subsequent waves (wave 3 to wave 6). Other variables that were independently and directly associated with increased likelihood of mortality were increasing age, current smoking, having heart disease, having dementia, having lung disease, and having cancer. Finally, older women had a decreased likelihood of mortality compared to older men.

**Table 2.** Direct Effects on All-Cause Mortality Risk and Recurrent Falls

Variable	Log-hazard odds	Hazard ratio	*SE*	Est/*SE*	*p* Value
All-cause mortality					
Visual difficulty^a^	0.392	1.48	0.100	3.935	.000
Recurrent falls^b^	0.391	1.48	0.083	4.696	.000
Age^c^	0.098	1.10	0.005	17.926	.000
Education^d^	0.046	1.05	0.144	0.318	.750
Female^e^	−0.456	0.63	0.074	−6.163	.000
Non-Hispanic Black^f^	0.306	1.36	0.237	1.289	.197
Hispanic^f^	0.228	1.26	0.242	0.942	.346
Non-Hispanic other^f^	−0.016	0.98	0.278	−0.058	.954
Healthy weight^g^	0.004	1.00	0.007	0.595	.552
Current smoking^h^	0.606	1.83	0.135	4.497	.000
Married^i^	0.134	1.14	0.080	1.682	.093
Heart disease	0.389	1.48	0.077	5.046	.000
hypertension	0.101	1.11	0.078	1.283	.200
Stroke	0.356	1.43	0.136	2.618	.009
Dementia	0.919	2.51	0.094	9.746	.000
Lung disease	0.342	1.41	0.082	4.196	.000
Cancer	0.286	1.33	0.091	3.148	.002
Hip fracture	−0.025	0.98	0.079	−0.316	.752
Medicare dual eligibility^j^	0.237	1.27	0.112	2.119	.034
Recurrent falls^b^					
Visual difficulty^a^	0.119	1.13	0.019	6.285	.000

*Notes*: *N* = 7,039. BMI = body mass index; Model fit: Loglikelihood: −4,842.8770; Akaike Information Criterion (AIC) = 10,117.754; Bayesian Information Criterion (BIC) = 11,597.354, sample-size adjusted BIC = 10,911.770. ref = reference; *SE* = standard error.

^a^ref: normal vision.

^b^ref: not experienced recurrent falls within 12 months.

^c^age in years.

^d^ref: high school degree/some college/undergraduate and graduate degree.

^e^ref: male.

^f^ref: non-Hispanic White.

^g^ref: underweight/overweight.

^h^ref: never smoked/former smoker.

^i^ref: divorced/separated/widowed/never married.

^j^ref: Medicare-only.

The results presented in Table 3 demonstrated a significant mediation effect of falls in the relationship between visual difficulty and mortality risk (*ab* = 0.046 (0.012), *p < .*001). The indirect effect of visual difficulty on mortality risk through falls showed a 0.046-unit increase in mortality risk. This suggests that in addition to the significant direct effect of visual difficulty on mortality risk (*c*’ = 0.422), there is also an indirect effect that accounts for 9.7% of the total effect (mediation proportion = 0.097).

## Discussion

Using data from the 2015–2020 NHATS and applying a pathway modeling approach, this study examined the association between visual difficulty, falls, and all-cause mortality among older adults. The results showed support for the direct effects of visual difficulty and recurrent falls on mortality risk, as well as for the mediation effect of recurrent falls.

This study contributes to the existing literature that has documented an association between vision loss and falls (Freeman et al., 2007; Jin et al., 2024; Legood et al., 2002). Although some studies reported inconsistent results regarding the association between vision loss and the incidence of falls among older adults (Freeman et al., 2007), findings from the current study supported the idea that older adults with visual difficulty are more likely to experience two or more falls in the 1-year observation period compared to those with normal vision. This was consistent with other studies. A review of the risks for and types of injuries associated with vision impairment indicated that individuals with vision loss were 1.7 times more likely to fall, and 1.9 times more likely to suffer recurrent falls (Legood et al., 2002). A recent study also reported that the association between low vision and recurrent falls was significant among both older males and older females (Singh & Maurya, 2022). One explanation is that visual difficulty is related to poor depth perception, or the ability to perceive objects in three dimensions, and monocular cues involving depth and motion signals (Harwood, 2001; Saftari & Kwon, 2018). For older adults, having visual difficulty can affect their balance control, lead to a reduced ability to perceive contrast, and make it difficult to respond to visual stimuli in the environment and coordinate movement (Lord & Dayhew, 2001), and impaired contrast sensitivity can result in a higher incidence of recurrent falls (Jin et al., 2024). Additionally, older people with visual difficulty may limit their activities due to a fear of falling (Ehrlich et al., 2019), which can further reduce their balance and strength. This lack of exercise and other physical activity increases the risk of falling up to threefold (Stokes, 2009).

This study also found an association between recurrent falls and increased risk of all-cause mortality among older adults. Studies using both clinical and nonclinical study designs assessed long-term survival in older adults who had experienced falls. However, findings were not always consistent and studies examining the mortality risk associated with recurrent falls are limited. However, an early study found an association between falling more than once and increased mortality at 1-year and 3-year follow-up among older adults living at home (Donald, 2002). Further, Sylliaas and colleagues (2009) reported that women living in the community who were aged 75 and older and who suffered at least two falls had poorer survival rates than nonfallers over 9 years of follow-up. Older adults in long-term care settings who fell twice in a year or more were also found to have a lower 5-year survival rate compared to those who fell once (Nurmi et al., 2004). A recent study conducted by Jónsdóttir and Ruthig (2021) reported that older adults who suffered a fall had poorer health outcomes and lower overall well-being, whereby experiencing one or more falls predicted significantly less likelihood of subsequent survival 7 years later. The evidence reported in the current study supports that experiencing more than one fall in the past put older adults at greater risk of mortality over the next few years.

Consistent with results from meta-analyses (Ehrlich et al., 2021; Zhang et al., 2016), the results from the current study showed that older adults with visual difficulty had a higher mortality risk than those without visual difficulty. Some studies suggested potential pathways that may explain the link between vision loss and mortality; however, these studies did not examine the role that falls play in this association. This study contributes to the existing literature by highlighting that recurrent falls mediate the link between visual difficulty and mortality.

In addition, these results provide evidence supporting the Stress Process Model. As a challenging chronic condition, visual difficulty is a stressor in one’s life and negatively influences health outcomes. Visual difficulty creates new strains, such as loss of balance control, difficulties in daily activities, limitations in functional abilities, less participation in physical activities, and decreases in independence and autonomy. Visual difficulty may also lead to the development of negative self-concepts, such as fear of falling and loss of self-esteem and mastery. Older adults with visual difficulty who experienced recurrent falls may be particularly vulnerable because falls as a stressful life event can amplify preexisting stressors by overwhelming people physically, creating more challenges in daily life, and preventing them from acquiring and employing coping resources. Older adults who experienced falls may suffer injuries, such as hip fractures or head trauma that lead to persistent impairments from which it is difficult to recover with advanced age. Falls and fall-related injuries can also decrease older adults’ independence and autonomy (Centers for Disease Control and Prevention, 2024), yielding a negative long-term impact on well-being and quality of life. According to the conceptualization of “stress proliferation” (Pearlin et al., 2005), falls caused by visual difficulty may, in turn, lead to more stress and worse outcomes, such as the increased risk of mortality. Given the effects of stressors on outcomes depend on the extent to which stressors proliferate (Pearlin et al., 1981), the combined stresses of visual difficulty and falls likely increase long-term mortality risk.

This study had several strengths. We employed DTSM based on a structural equation modeling framework to examine the pathways and mechanisms for the association between visual difficulty and mortality risk among a national U.S. sample of older adults. Using these models, our results confirmed there is a risk of underestimating the overall impact of visual difficulty on mortality risk when attention is confined to direct effects. In future research, it will be worthwhile to extend the current DTSM model to investigate joint event histories and growth processes that involve time-varying indicators. In addition to the current findings, we estimated the same models with a time-varying mediator (recurrent falls at wave 2 and wave 3). The results showed that visual difficulty at baseline was significantly associated with mortality risk (β (log-hazard odds) = 0.359 (0.100), *p* < .001), and the indirect effect of falls remained statistically significant (β = 0.076 (0.016, *p* < .001)), with a 17.5% mediation effect. There were significant indirect effects through falls at wave 2 alone (β = 0.027 (0.012), *p* < .05), at wave 3 alone (β = 0.029 (0.011), *p* < .01), as well as through wave 2 falls first and wave 3 falls second (β = 0.020 (0.006), *p < .*001). Because falls at wave 2 were strongly associated with falls at wave 3 (β = 0.436 (0.019), *p < *.001), this suggests prior falls as the risk factor for subsequent falls and the importance of the cumulative effect of falls across time. In addition, the cumulative effects of recurrent falls may be a marker of other underlying causes of increased mortality risk, such as frailty. The impact of visual difficulty on mortality may operate through complex, time-dependent pathways. Future studies should further explore the cumulative effect of falls across time as well as the temporal dynamics between falls and mortality for older adults with visual difficulty, to better understand these mechanisms.

### Implications

The findings of this study have significant practical implications for public health, policy development, and future research. Older adults and others involved in the care of older adults should be aware of the risks of recurrent falls, especially for those with visual difficulty. Research indicates that falls are not solely caused by a hazardous environment (Buckley et al., 2005); rather, an individual’s capacity to navigate an unsafe environment is a crucial factor, which is influenced by the interplay between the person and their surroundings. Older adults with visual difficulty may struggle to manage an unsafe environment and they may not be able to detect hazards optimally, which could result in falls. Regular eye exams are crucial for early detection and management of eye diseases like cataracts, glaucoma, and AMD. Policies that improve access to affordable, regular eye care for older adults, particularly those in underserved communities, could have a profound impact on fall prevention efforts. Furthermore, research indicates that some older adults with vision loss who experienced falls report they had no choice but to perform essential household tasks on their own, even though they knew it could put them at risk of falling (Brundle et al., 2015). For some older adults, it may be beneficial to have a thorough assessment of their living environment and their subsequent needs to understand whether they lack support and whether there are ways to increase their safety and well-being. Fall prevention programs that include exercise regimens designed to improve balance, strength, and coordination are particularly beneficial for individuals with vision loss. Community-based programs like tai chi or physical therapy sessions could help enhance functional mobility and reduce fall risk. Ensuring that these programs are accessible and inclusive for those with visual difficulty is essential.

From a research perspective, future studies should evaluate the effectiveness of targeted interventions and explore how they can be tailored to older adults with visual difficulty. Expand our understanding of how social support influences the relationship between vision loss, falls, and mortality is essential. Instrumental support, such as helping older adults use assistive devices and rehabilitative services, can enhance their adjustment to vision loss and reduce fall-related risks. Informational support, by offering strategies to interact safely with the environment, also plays a key role in prevention. Future research should investigate how social support can prevent future falls, aid recovery, and improve quality of life and survival among older adults with visual difficulty who have experienced falls. Additionally, addressing systemic barriers, such as disparities in healthcare access and affordability, is vital to reducing the overall burden of falls in this population.

### Limitations

This study also has limitations. One limitation of the current study was that visual difficulty was measured at baseline only. It is possible that some participants developed visual difficulty in the following waves. Individuals with long-term visual difficulties may also have developed skills or coping strategies to prevent falls and related injuries. Future research should expand on the research design employed in this study. Additionally, the measure of vision in our study is based on the participant’s self-reported vision status and does not represent an objective measure of visual function. Future research should investigate the impact of duration of vision loss on falls and mortality risk using an objective assessment of visual function. Moreover, researchers have observed that individuals who experience recurrent falls are more likely to be transferred to skilled nursing care units in nursing homes (Gaebler, 1993). As a result, some participants who were admitted to long-term care settings were not included in our study, which could lead to survival bias. It is also possible that improvements in management practices for falls in some long-term care settings over the last decade may result in improved survival rates. In addition, information regarding falls was collected through self-reporting, where participants were asked if they had experienced multiple falls in the past year. Recall may be greater over a long period of time. It is possible that the occurrence of multiple falls was underreported. Finally, due to the limitations of the measures of physical activity in the NHATS, we did not evaluate this potential pathway between vision loss and mortality. Future research should build upon our study by examining physical activity as a mediator.

## Conclusion

Using a large national data source, this study contributed to the scientific literature by demonstrating that adults with visual difficulty are at a higher risk of all-cause mortality, and that falls play a crucial role in this association. The findings highlighted the importance of promoting interventions to decrease the risk of falls among the aging population and, in particular, for those who experience vision loss. Further research is necessary to examine other factors that modify and mediate the relationship between vision loss and mortality.

## Supplementary Material

igaf016_suppl_Supplementary_Figure_S1

## Data Availability

This study was not preregistered. The data used in this study are from the National Health and Aging Trends Study (NHATS), which is publicly available through the NHATS website (https://www.nhats.org/researcher/nhats).

## References

[CIT0001] Aneshensel, C. S., & Mitchell, U. A. (2014). The stress process: Its origins, evolution, and future. In R. J. Johnson, R. J. Turner, & B. G. Link (Eds.), Sociology of mental health: Selected topics from forty years 1970s-2010s (pp. 53–74). Springer.

[CIT0002] Berg, R. L., & Cassells, J. S. (1992). Falls in older persons: Risk factors and prevention. In R. L. Berg & J. S. Cassells (Eds.), The second fifty years: Promoting health and preventing disability. National Academies Press. 25144081

[CIT0003] Brundle, C., Waterman, H. A., Ballinger, C., Olleveant, N., Skelton, D. A., Stanford, P., & Todd, C. (2015). The causes of falls: Views of older people with visual impairment. Health Expectations, 18(6), 2021–2031. https://doi.org/10.1111/hex.12355 25736829 PMC4949546

[CIT0004] Buckley, J. G., Heasley, K. J., Twigg, P., & Elliott, D. B. (2005). The effects of blurred vision on the mechanics of landing during stepping down by the elderly. Gait & Posture, 21(1), 65–71. https://doi.org/10.1016/j.gaitpost.2003.12.001 15536035

[CIT0005] Buckley, J. G., Panesar, G. K., MacLellan, M. J., Pacey, I. E., & Barrett, B. T. (2010). Changes to control of adaptive gait in individuals with long-standing reduced stereoacuity. Investigative Ophthalmology & Visual Science, 51(5), 2487–2495. https://doi.org/10.1167/iovs.09-3858 20335609

[CIT0006] Burns, E., & Kakara, R. (2018). Deaths from falls among persons aged ≥65 years - United States, 2007-2016. Morbidity and Mortality Weekly Report, 67(18), 509–514. https://doi.org/10.15585/mmwr.mm6718a1 29746456 PMC5944976

[CIT0007] Centers for Disease Control and Prevention. (2024). Facts about falls. https://www.cdc.gov/falls/data-research/facts-stats/index.html

[CIT0008] Christ, S. L., Lee, D. J., Lam, B. L., Zheng, D. D., & Arheart, K. L. (2008). Assessment of the effect of visual impairment on mortality through multiple health pathways: Structural equation modeling. Investigative Ophthalmology & Visual Science, 49(8), 3318–3323. https://doi.org/10.1167/iovs.08-1676 18362104

[CIT0009] Crews, J. E., Chou, C.-F., Stevens, J. A., & Saaddine, J. B; DPA. (2016). Falls among persons aged ≥65 years with and without severe vision impairment—United States, 2014. Morbidity and Mortality Weekly Report, 65(17), 433–437. https://doi.org/10.15585/mmwr.mm6517a2 27148832

[CIT0010] Dhital, A., Pey, T., & Stanford, M. R. (2010). Visual loss and falls: A review. Eye, 24(9), 1437–1446. https://doi.org/10.1038/eye.2010.60 20448666

[CIT0011] Ditlevsen, S., Christensen, U., Lynch, J., Damsgaard, M. T., & Keiding, N. (2005). The mediation proportion: A structural equation approach for estimating the proportion of exposure effect on outcome explained by an intermediate variable. Epidemiology, 16(1), 114–120. https://doi.org/10.1097/01.ede.0000147107.76079.07 15613954

[CIT0055] Donald, I. (2002). The prognosis of falls in elderly people living at home. Age and Ageing, 28(2), 121–125. https://doi.org/10.1093/ageing/28.2.121 10350407

[CIT0012] Ehrlich, J. R., Hassan, S. E., & Stagg, B. C. (2019). Prevalence of falls and fall-related outcomes in older adults with self‐reported vision impairment. Journal of the American Geriatrics Society, 67(2), 239–245. https://doi.org/10.1111/jgs.15628 30421796 PMC6367006

[CIT0013] Ehrlich, J. R., Ramke, J., Macleod, D., Burn, H., Lee, C. N., Zhang, J. H., Waldock, W., Swenor, B. K., Gordon, I., Congdon, N., Burton, M., & Evans, J. R. (2021). Association between vision impairment and mortality: A systematic review and meta-analysis. Lancet Global Health, 9(4), e418–e430. https://doi.org/10.1016/S2214-109X(20)30549-0 33607015 PMC7966688

[CIT0014] Fairchild, A. J., Abara, W. E., Gottschall, A. C., Tein, J. Y., & Prinz, R. J. (2015). Improving our ability to evaluate underlying mechanisms of behavioral onset and other event occurrence outcomes: A discrete-time survival mediation model. Evaluation & the Health Professions, 38(3), 315–342. https://doi.org/10.1177/0163278713512124 24296470 PMC4594798

[CIT0015] Folkman, S. (2012). Stress, coping, and hope. In B. Carr & J. Steel (Eds.), Psychological aspects of cancer (pp. 119–127). Springer. https://doi.org/10.1002/pon.1836

[CIT0016] Forman-Hoffman, V. L., Ault, K. L., Anderson, W. L., Weiner, J. M., Stevens, A., Campbell, V. A., & Armour, B. S. (2015). Disability status, mortality, and leading causes of death in the United States community population. Medical Care, 53(4), 346–354. https://doi.org/10.1097/MLR.0000000000000321 25719432 PMC5302214

[CIT0017] Freeman, E. E., Munoz, B., Rubin, G., & West, S. K. (2007). Visual field loss increases the risk of falls in older adults: The Salisbury eye evaluation. Investigative Ophthalmology & Visual Science, 48(10), 4445–4450. https://doi.org/10.1167/iovs.07-0326 17898264

[CIT0018] Gaebler, S. (1993). Predicting which patient will fall again... and again. Journal of Advanced Nursing, 18(12), 1895–1902. https://doi.org/10.1046/j.1365-2648.1993.18121895.x 8132920

[CIT0019] Gupta, P., Man, R. E. K., Fenwick, E. K., Qian, C., Sim, R., Majithia, S., Tham, Y. C., Sabanayagam, C., Wong, T. Y., Cheng, C. Y., & Lamoureux, E. L. (2023). Associations between visual impairment, incident falls and fall frequency among older Asians: Longitudinal findings from the Singapore Epidemiology of Eye Diseases study. British Journal of Ophthalmology, 107(11), 1590–1596. https://doi.org/10.1136/bjo-2021-320873 35914927

[CIT0020] Guralnik, J. M., Ferrucci, L., Simonsick, E. M., Salive, M. E., & Wallace, R. B. (1995). Lower-extremity function in persons over the age of 70 years as a predictor of subsequent disability. New England Journal of Medicine, 332(9), 556–561. https://doi.org/10.1056/NEJM199503023320902 7838189 PMC9828188

[CIT0021] Harwood R. H. (2001). Visual problems and falls. Age and Ageing, 30(Suppl 4), 13–18. https://doi.org/10.1093/ageing/30.suppl_4.13 11769782

[CIT0022] Heron, M. P. (2017). Deaths: Leading causes for 2015. National Vital Statistics Reports. https://stacks.cdc.gov/view/cdc/50010 29235984

[CIT0023] Hudak, K. M. A., Wittenborn, J. S., Lamuda, P. A., Lundeen, E. A., Saaddine, J., & Rein, D. B. (2023). Association between social determinants of health and examination-based vision loss vs self-reported vision measures. JAMA Ophthalmology, 141(5), 468–476. https://doi.org/10.1001/jamaophthalmol.2023.0723 37022712 PMC10080399

[CIT0024] James, S. L., Lucchesi, L. R., Bisignano, C., Castle, C. D., Dingels, Z. V., Fox, J. T., Hamilton, E. B., Henry, N. J., Krohn, K. J., Liu, Z., McCracken, D., Nixon, M. R., Roberts, N. L. S., Sylte, D. O., Adsuar, J. C., Arora, A., Briggs, A. M., Collado-Mateo, D., Cooper, C., … Murray C. J. L. (2020). The global burden of falls: Global, regional and national estimates of morbidity and mortality from the Global Burden of Disease Study 2017. Injury Prevention, 26(Supp 1), i3–i11. https://doi.org/10.1136/injuryprev-2019-043286 31941758 PMC7571347

[CIT0025] Jin, H., Zhou, Y., Stagg, B. C., & Ehrlich, J. R. (2024). Association between vision impairment and increased prevalence of falls in older US adults. Journal of the American Geriatrics Society, 72(5), 1373–1383. https://doi.org/10.1111/jgs.18879 38514075 PMC11090722

[CIT0026] Jónsdóttir, H. L., & Ruthig, J. C. (2021). A longitudinal study of the negative impact of falls on health, well-being, and survival in later life: The protective role of perceived control. Aging & Mental Health, 25(4), 742–748. https://doi.org/10.1080/13607863.2020.1725736 32081033

[CIT0027] Kasper, J. D., Freedman, V. A., & Spillman, B. C. (2013). Classification of persons by dementia status in the National Health and Aging Trends Study. Technical Paper, 5, 1–4.

[CIT0028] Klein, R., Lee, K. E., Gangnon, R. E., & Klein, B. E. (2014). Relation of smoking, drinking, and physical activity to changes in vision over a 20-year period: The Beaver Dam Eye Study. Ophthalmology, 121(6), 1220–1228. https://doi.org/10.1016/j.ophtha.2014.01.003 24594095 PMC4047137

[CIT0029] Legood, R., Scuffham, P., & Cryer, C. (2002). Are we blind to injuries in the visually impaired? A review of the literature. Injury Prevention, 8(2), 155–160. https://doi.org/10.1136/ip.8.2.155 12120837 PMC1730864

[CIT0030] Lord, S. R., & Dayhew, J. (2001). Visual risk factors for falls in older people. Journal of the American Geriatrics Society, 49(5), 508–515. https://doi.org/10.1046/j.1532-5415.2001.49107.x 11380741

[CIT0031] McDaniel, H. L. (2018). Advancing understanding of dynamic mechanisms in onset to event models: Discrete time survival mediation with a time variant mediator [Doctoral dissertation]. University of South Carolina. https://scholarcommons.sc.edu/etd/4673/

[CIT0032] Mehta, J., Czanner, G., Harding, S., Newsham, D., & Robinson, J. (2022). Visual risk factors for falls in older adults: A case-control study. BMC Geriatrics, 22(1), 134. https://doi.org/10.1186/s12877-022-02784-3 35177024 PMC8855581

[CIT0033] Muthén, B., & Masyn, K. (2005). Discrete-time survival mixture analysis. Journal of Educational and Behavioral Statistics, 30(1), 27–58. https://doi.org/10.3102/10769986030001027

[CIT0034] Nurmi, I. S., Lüthje, P. M., & Kataja, J. M. (2004). Long-term survival after falls among the elderly in institutional care. Archives of Gerontology and Geriatrics, 38(1), 1–10. https://doi.org/10.1016/s0167-4943(03)00079-7 14599699

[CIT0035] Padrón-Monedero, A., Pastor-Barriuso, R., García López, F. J., Martínez Martín, P., & Damián, J. (2020). Falls and long-term survival among older adults residing in care homes. PloS One, 15(5), e0231618. https://doi.org/10.1371/journal.pone.0231618 32379771 PMC7205288

[CIT0036] Panel on Prevention of Falls in Older Persons, American Geriatrics Society and British Geriatrics Society. (2011). Summary of the Updated American Geriatrics Society/British Geriatrics Society clinical practice guideline for prevention of falls in older persons. Journal of the American Geriatrics Society, 59(1), 148–157. https://doi.org/10.1111/j.1532-5415.2010.03234.x 21226685

[CIT0037] Patel, N., Stagg, B. C., Swenor, B. K., Zhou, Y., Talwar, N., & Ehrlich, J. R. (2020). Association of co-occurring dementia and self-reported visual impairment with activity limitations in older adults. JAMA Ophthalmology, 138(7), 756–763. https://doi.org/10.1001/jamaophthalmol.2020.1562 32407444 PMC7226290

[CIT0038] Pearl J. (2014). Interpretation and identification of causal mediation. Psychological Methods, 19(4), 459–481. https://doi.org/10.1037/a0036434 24885338

[CIT0039] Pearlin, L. I., & Bierman, A. (2013). Current issues and future directions in research into the stress process. In C. S. Aneshensel, J. C. Phelan, & A. Bierman (Eds.), Handbook of the sociology of mental health (pp. 325–340). Springer. https://doi.org/10.1007/978-94-007-4276-5_16

[CIT0040] Pearlin, L. I., Menaghan, E. G., Lieberman, M. A., & Mullan, J. T. (1981). The stress process. Journal of Health and Social Behavior, 337–356. https://doi.org/10.2307/2136676 7320473

[CIT0041] Pearlin, L. I., Schieman, S., Fazio, E. M., & Meersman, S. C. (2005). Stress, health, and the life course: Some conceptual perspectives. Journal of Health and Social Behavior, 46(2), 205–219. https://doi.org/10.1177/002214650504600206 16028458

[CIT0042] Pratschke, J., Haase, T., Comber, H., Sharp, L., de Camargo Cancela, M., & Johnson, H. (2016). Mechanisms and mediation in survival analysis: Towards an integrated analytical framework. BMC Medical Research Methodology, 16, 27. https://doi.org/10.1186/s12874-016-0130-6 26927506 PMC4772586

[CIT0043] Ramulu, P. Y., Mihailovic, A., West, S. K., Gitlin, L. N., & Friedman, D. S. (2019). Predictors of falls per step and falls per year at and away from home in glaucoma. American Journal of Ophthalmology, 200, 169–178. https://doi.org/10.1016/j.ajo.2018.12.021 30639366 PMC6445769

[CIT0044] Rubenstein L. Z. (2006). Falls in older people: Epidemiology, risk factors and strategies for prevention. Age and Ageing, 35(Suppl 2), ii37–ii41. https://doi.org/10.1093/ageing/afl084 16926202

[CIT0045] Saftari, L. N., & Kwon, O. S. (2018). Ageing vision and falls: A review. Journal of Physiological Anthropology, 37(1), 11. https://doi.org/10.1186/s40101-018-0170-1 29685171 PMC5913798

[CIT0046] Schubert, C. R., Fischer, M. E., Pinto, A. A., Klein, B. E., Klein, R., Tweed, T. S., & Cruickshanks, K. J. (2017). Sensory impairments and risk of mortality in older adults. Journals of Gerontology, Series A: Biomedical Sciences and Medical Sciences, 72(5), 710–715. https://doi.org/10.1093/gerona/glw036 PMC5861964 26946102

[CIT0047] Singer, J. D., & Willett, J. B. (2003). Applied longitudinal data analysis: Modeling change and event occurrence. Oxford University Press.

[CIT0056] Singh, R. R., & Maurya, P. (2022). Visual impairment and falls among older adults and elderly: evidence from longitudinal study of ageing in India. BMC Public Health, 22(1). https://doi.org/10.1186/s12889-022-14697-2 PMC9746100 36510173

[CIT0048] Sloan, F. A., Ostermann, J., Brown, D. S., & Lee, P. P. (2005). Effects of changes in self-reported vision on cognitive, affective, and functional status and living arrangements among the elderly. American Journal of Ophthalmology, 140(4), 618–627. https://doi.org/10.1016/j.ajo.2005.01.019 16226514

[CIT0049] Stokes, J. M. (2009), Falls in older people: Risk factors and strategies for prevention. Australasian Journal on Ageing, 28, 47–47. https://doi.org/10.1111/j.1741-6612.2009.00347.x

[CIT0050] Sylliaas, H., Idland, G., Sandvik, L., Forsen, L., & Bergland, A. (2009). Does mortality of the aged increase with the number of falls? Results from a nine-year follow-up study. European Journal of Epidemiology, 24(7), 351–355. https://doi.org/10.1007/s10654-009-9348-5 19452127

[CIT0051] Vaishya, R., & Vaish, A. (2020). Falls in older adults are serious. Indian Journal of Orthopaedics, 54(1), 69–74. https://doi.org/10.1007/s43465-019-00037-x 32257019 PMC7093636

[CIT0054] Winter, J. E., MacInnis, R. J., Wattanapenpaiboon, N., & Nowson, C. A. (2014). BMI and all-cause mortality in older adults: a meta-analysis. The American Journal of Clinical Nutrition, 99(4), 875–890. https://doi.org/10.3945/ajcn.113.068122 24452240

[CIT0052] Zhang, T., Jiang, W., Song, X., & Zhang, D. (2016). The association between visual impairment and the risk of mortality: A meta-analysis of prospective studies. Journal of Epidemiology and Community Health, 70(8), 836–842. https://doi.org/10.1136/jech-2016-207331 27095181

[CIT0053] Zheng, D. D., Christ, S. L., Lam, B. L., Arheart, K. L., Galor, A., & Lee, D. J. (2012). Increased mortality risk among the visually impaired: The roles of mental well-being and preventive care practices. Investigative Ophthalmology & Visual Science, 53(6), 2685–2692. https://doi.org/10.1167/iovs.11-8794 22427599 PMC3366723

