# Supplementary material for: Self-Reported Visual Difficulty and Mortality Risk Among Older Adults: The Mediating Role of Recurrent Falls
Source: Innov Aging. 2025 Feb 17;9(5):igaf016. doi: 10.1093/geroni/igaf016 (PMC12082091; doi:10.1093/geroni/igaf016)
Supplement: igaf016_suppl_Supplementary_Figure_S1 [file igaf016_suppl_supplementary_figure_s1.docx]

***Innovation in Aging* Supplementary Material: Xu, Burr, Song, & Ehrlich. Self-Reported Visual Difficulty and Mortality Risk Among Older Adults: The Mediating Role of Recurrent Falls.**

**Supplementary Figure 1.** Kaplan-Meier Curves of 5-Year Survival


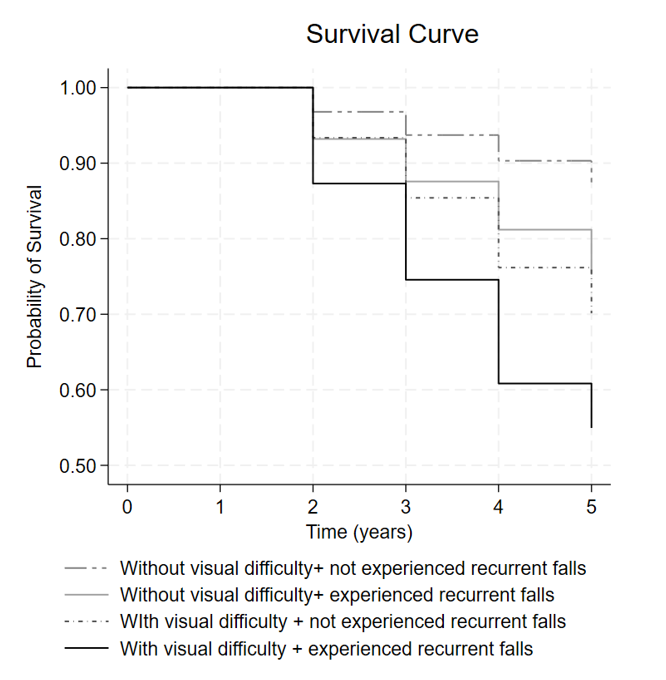


*Note.* Kaplan-Meier curves displaying the overall survival probability among older adults stratified by visual difficulty and recurrent falls.
